# Non-Invasive Electromagnetic Skin Patch Sensor to Measure Intracranial Fluid–Volume Shifts

**DOI:** 10.3390/s18041022

**Published:** 2018-03-29

**Authors:** Jacob Griffith, Kim Cluff, Brandon Eckerman, Jessica Aldrich, Ryan Becker, Peer Moore-Jansen, Jeremy Patterson

**Affiliations:** 1 Biomedical Engineering, Wichita State University, Wichita, KS 67260, USA; jlgriffith2@shockers.wichita.edu (J.G.); bdeckerman@shockers.wichita.edu (B.E.); jxaldrich@shockers.wichita.edu (J.A.); linkxor@gmail.com (R.B.); 2 Department of Anthropology, Wichita State University, Wichita, KS 67260, USA; pmojan@wichita.edu; 3 Human Performance Studies, Wichita State University, Wichita, KS 67260, USA; jeremy.patterson@wichita.edu; 4 Institute of Interdisciplinary Creativity, Wichita State University, Wichita, KS 67260, USA

**Keywords:** dielectrics, health monitoring systems, intracranial pressure sensors, microwave sensors, point-of-care technologies, volume measurement, wearable sensors

## Abstract

Elevated intracranial fluid volume can drive intracranial pressure increases, which can potentially result in numerous neurological complications or death. This study’s focus was to develop a passive skin patch sensor for the head that would non-invasively measure cranial fluid volume shifts. The sensor consists of a single baseline component configured into a rectangular planar spiral with a self-resonant frequency response when impinged upon by external radio frequency sweeps. Fluid volume changes (10 mL increments) were detected through cranial bone using the sensor on a dry human skull model. Preliminary human tests utilized two sensors to determine feasibility of detecting fluid volume shifts in the complex environment of the human body. The correlation between fluid volume changes and shifts in the first resonance frequency using the dry human skull was classified as a second order polynomial with *R*^2^ = 0.97. During preliminary and secondary human tests, a ≈24 MHz and an average of ≈45.07 MHz shifts in the principal resonant frequency were measured respectively, corresponding to the induced cephalad bio-fluid shifts. This electromagnetic resonant sensor may provide a non-invasive method to monitor shifts in fluid volume and assist with medical scenarios including stroke, cerebral hemorrhage, concussion, or monitoring intracranial pressure.

Jessica Aldrich ^1^

## 1. Introduction

Pathological increases in intracranial pressure (ICP) can be associated with a number of neurological complications and even death in patients who have had stroke, traumatic brain injury, inflammatory response of the central nervous system, or have undergone neurosurgical or neurological procedures. These complications can include cerebral hemorrhage, stroke, and irreparable brain damage associated with increased morbidity and mortality [1,2]. Variations in ICP are driven by fluid volume changes of the primary components within the cranial cavity; these components include brain tissue, blood, and cerebrospinal fluid (CSF) [2]. Equilibrium in the combined volume of these components maintains an equilibrium in ICP allowing constant pressure. Pressure in the human skull is regulated and maintained by autoregulation mechanisms which allow cerebrospinal fluid shifts from the ventricles of the brain to the spinal column. In most individuals, a compensatory reserve exists that allows for increases of 60 mL to 80 mL in intracranial volume with minimal pressure increases [1]. When the autoregulation mechanisms are disrupted, and excessive fluid volumes persist in the cranial cavity, ICP increases exponentially due to the volume–pressure relationship being non-linear [1,2,3,4].

Currently, ICP is measured primarily through invasive methods, including intraventricular catheters, external ventricular drains, lumbar puncture, and micro transducer ICP monitoring devices. External ventricular drains are the gold standard, as they allow for drainage of excess CSF from the ventricles. Complications and limitations of this method include infection, hemorrhaging, and the need of a neurosurgeon in a clinical setting [1,2,3,5]. Clinical situations requiring an ICP measurement in which an invasive procedure is contraindicated highlight the need for an improved non-invasive ICP measurement method. Through the use of a non-invasive ICP measurement, treatments can be optimized for patients with conditions such as fulminant hepatic failure and preeclampsia where invasive ICP measurement is contraindicated [6]. An additional clinical advantage of monitoring the cerebrovascular function through non-invasive assessment of intracranial fluid volume is the ability to screen for intracranial bleeding, impaired CSF reabsorption, and assess severity of injury following brain trauma [7]. Another situation requiring non-invasive assessment of either ICP or intracranial fluid volume arises when astronauts are exposed to a microgravity environment during long duration spaceflight missions. Monitoring of CSF and cerebral blood volume is an area of interest concerning long duration spaceflight missions due to its potential impact on astronaut crew health [3,8,9]. However, sufficient quantitative data has not been obtained to conclusively determine the role of elevated ICP on human physiology in a microgravity environment [8]. Overall, these situations highlight the need for development of non-invasive, point-of-care technologies to monitor intracranial fluid volume.

Investigations into the development of non-invasive methods to measure ICP and intracranial volume have included transcranial Doppler ultrasonography (TCD), magnetic resonance imaging (MRI), computed tomography (CT), optic nerve sheath diameter (ONSD) measurement, and tympanic membrane displacement [1,3]. These methods eliminate the risk of infection and hemorrhaging, but they can be limited in accuracy, require expensive specialized equipment, and cannot be used in a point-of-care setting [1,3]. Ultrasonography is highly appealing, however, ultrasound waves do not adequately penetrate bone, making measurement locations limited to cranial suture joints which require precise positioning of the transducer [3]. Attempts to increase the accuracy of ICP estimates with TCD using data mining have been explored, but with limited success [6].

### State of the Art in Intracranial Pressure Measurement

Recent advancements regarding the non-invasive measurement of intracranial fluid level shifts have focused upon the use of electrical impedance spectroscopy, magnetic induction spectroscopy, and volumetric inductive phase shift spectroscopy [10,11,12,13,14,15,16,17,18,19]. While these non-invasive methods have promise, they are lacking in simplicity and form factor suitable for wearable, point-of-care measurements. Current electromagnetic tomography imaging systems exist, but utilize multiple antennae that alternate between transmitting and receiving signals to create the image. 

In contrast, open circuit resonant sensors, which utilize operating principles similar to electromagnetic tomography imaging systems, are more simplistic in design and form factor. Open circuit resonant sensors can be designed as wearable, soft bioelectronics, much like a skin patch that can be applied as an adhesive bandage, allowing it to be used as a point-of-care diagnostic technology. Open circuit resonant sensors do not require electrical components and are passively energized using an external radiofrequency (RF) sweep. Based upon the premise that impedance fluctuations arise as a result of fluid volume changes, open circuit electromagnetic skin patch sensors may be able to detect changes in intracranial fluid volume levels [14,20]. Recent advances in research have improved the development and optimization of open circuit resonant sensors [21,22,23,24]. These advances have also been implemented in our previous work to measure limb hemodynamics, detect changes in volume, and obtain usable sensor signal response regarding fluid filling in the left ventricle of a bovine heart [20,21,25,26,27].

The focus of this study was to evaluate changes in the resonant frequency as a result of volumetric shifts of intracranial fluids using an open circuit electromagnetic resonant skin patch sensor. It is hypothesized that fluid volume changes inside a human skull can be detected as a shift in the resonant frequency of the open circuit resonant sensor skin patch. The objectives of this study were (1) to develop a point-of-care, non-invasive electromagnetic resonant skin patch sensor to measure changes in intracranial fluid volume; (2) to detect fluid volume shifts inside a dry human skull model; and (3) to obtain usable sensor signal response associated with induced cephalad bio-fluid shifts in preliminary, proof-of-concept human tests.

## 2. Materials and Methods

### 2.1. Sensor Design and Data Collection Setup

**Figure 1:**
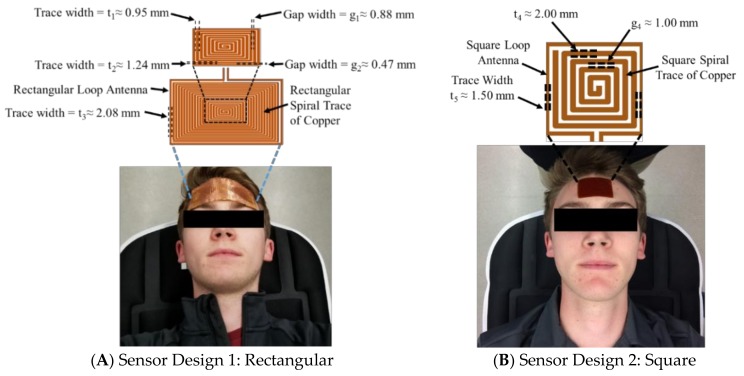
Electromagnetic skin patch sensors developed to measure changes in intracranial fluid volume. Data was collected from six human participants (following Institutional Review Board (IRB) approved protocol).

The electromagnetic resonant sensor patch was designed and built from a single baseline component: a trace of copper which was configured into a rectangular planar spiral (Figure 1A). Two separate designs were utilized in this study. The rectangular spiral patch (11.83 cm × 5.92 cm) had 20 turns, a trace width (*t*_1_, along the minor axis) of 0.95 mm and a gap width (*g*_1_, along the minor axis) of 0.88 mm, and a trace width (*t*_2_, along the major axis) of approximately 1.24 mm and a gap width (*g*_2_, along the major axis) of 0.47 mm. To address the large size and bulky form factor of the first sensor’s design, a second patch was designed as a square planar spiral (Figure 1B) to reduce surface area, increase ease of adherence to the forehead, and possess a more wearable form factor for human testing. This square sensor design (2.8 cm × 2.8 cm) had 5 turns, a trace width (*t*_4_) of 2.00 mm and a gap width (*g*_4_) of 1.00 mm. 

In order to measure shifts in the resonant frequency response of the rectangular sensor design, a rectangular loop antenna (12.34 cm × 6.5 cm) surrounding the sensor with a trace width (*t*_3_) of 2.08 mm was connected to a coaxial cable using a 50 Ohm SMA (SubMiniature version A) connector. The square sensor design utilized a similar set-up, however, the square loop antenna (3.38 cm × 3.38 cm) surrounding the sensor had a trace width (*t*_5_) of 1.50 mm. In both set-ups, the antenna produced the radiofrequency (RF) wave that interrogated and became electromagnetically coupled with the sensor. A vector network analyzer (VNA) (R&S ZNC-3) was used to produce an RF sweep in the desired frequency range and measure the return loss S-parameter, or the S_11_ reflection coefficient. Prior to data collection, the VNA was calibrated using the open short match (OSM) method over the desired frequency bandwidth.

### 2.2. Theory and Operating Principle

Incident RF waves, originating from the antenna, interrogate the electromagnetic resonant patch sensor which induces a current in the copper trace. In accordance to Maxwell’s equations of electric and magnetic fields and the right hand rule, oscillating electric and magnetic fields are formulated around the sensor [23]. The magnitude of the magnetic field is dependent upon the inductance (*L*) value, which can be calculated using Equation (1) [23,28].

(1) $$L=\frac{\mu_{0}}{4\pi I^{2}}\iint \left[\frac{J(r_{i})\mu_{i}*J(r_{j})\mu_{j}}{\left|r_{i}-r_{j}\right|}\right]\;d^{3}r_{i}d^{3}r_{j}$$

where, *L* is the total inductance, *J*(*r_i_*) is the spatial current density as a function of *r_i_* which is the length of the sensor trace, *µ*_0_ is the free space magnetic permeability, *µ_i_* is the relative magnetic permeability, and *I* is the total current in the circuit.

In addition, the gaps between traces provide parasitic capacitance with an electric field being developed between the traces during times of resonance. Resonance occurs at certain frequencies when energy is alternatively stored in magnetic and electric fields [28]. The ability of the electric and magnetic fields to develop depends on the electric permittivity and magnetic permeability of the substrate. The capacitance value of the sensor can be calculated using Equation (2) [23,28].

(2) $$C^{-1}=\;\frac{1}{4\varepsilon_{0}Q^{2}}\iint \;\left[\frac{\rho \left(r_{i}\right)\rho \left(r_{j}\right)k_{ij}}{\left|r_{i}-r_{j}\right|}\right]d^{3}r_{i}d^{3}r_{j}$$

where, *C* is the capacitance, *ρ*(*r_i_*) is the spatial charge density as function of *r_i_* which is the length of the sensor trace, *ε*_0_ is the free space electrical permittivity, *k_ij_* is the relative permittivity, and *Q* is the total charge density.

Alterations of the sensor design parameters, including size, shape, number of turns in the inductor planar spiral, trace width of coil, and gap between traces, results in a unique electromagnetic field. Each sensor’s electromagnetic field will consistently have a specific magnitude while the resonant frequency response is substrate specific. The first principal resonant frequency can be calculated using Equation (3).

(3) $$f=\frac{1}{2\pi \sqrt{LC}}$$

where, *f* is the first principal resonant frequency, *L* is the inductance, and *C* is the capacitance.

Electromagnetic sensors detect volumetric changes in layered material as shifts in the resonant frequency due to changes in the effective permittivity [28,29]. In regards to non-invasive electromagnetic intracranial fluid shift detection, the substrate composition of blood, brain tissue, and cerebrospinal fluid in the cranial cavity act as a layered substrate material. When the fluid volume changes in these layers, the effective permittivity of the substrate interacting with the electromagnetic field of the resonant sensor produces a change in the resonant equivalent inductance and capacitance causing a shift in the resonant frequency response. Thus, changes in the substrate, such as an increase in blood volume without a corresponding CSF decrease, are associated with shifts in the effective electric permittivity which is detected using the sensor and quantified through the S_11_ reflection coefficient. In our previous work [21], we further outlined the theory and operating principles.

In regards to safety, the power output from the VNA we used for the study was very low (at 0 dBm). One way to quantify the safety aspects is through specific absorption rate (SAR). SAR is an important measure used to assess whether an RF wave emitting device is safe for use near or in the human body [30]. Specific absorption rate (SAR) measurement of the sensor was conducted on the head for an in vivo, non-invasive, on-the-body measurement to determine the power absorbed into the tissue and subsequently calculate the SAR value using the method described in the literature [31].

### 2.3. Dry Human Skull Model

**Figure 2:**
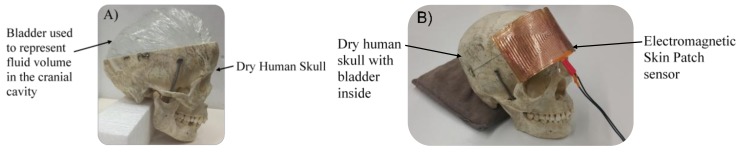
(**A**) A dry human skull and bladder filled with water were used to represent fluid volume changes in the cranial cavity; (**B**) The sensor was placed on the outside of the skull and a syringe with tube was used to incrementally simulate a fluid volume shift in the cranium.

The pressure–volume relationship inside the cranial cavity dictates that pathological increases in intracranial pressure are driven by a volume increase of blood, cerebrospinal fluid, or brain tissue. Non-invasively detecting shifts in intracranial pressure requires a sensor that is able to detect fluid volume changes through cranial bone and result in a unique sensor signal response. A physical model was constructed to test the correlation between signal response and fluid volume changes. Using a dry human skull, a bladder was inserted into the cranium and filled with 800 mL of water (*ε_r_* ≈ 78) to represent the intracranial fluid volume (Figure 2). Additional fluid was added in increments of 10 mL using a syringe and plastic tubing. Fluid volume increments of 10 mL were used to determine the sensor’s ability to detect volumetric changes less than the compensatory reserve (60–80 mL). Volumetric shifts exceeding the compensatory reserve are necessary for a significant pressure increase. Therefore, the detection of a volume fluctuation greater than the compensatory reserve may be used to detect a significant ICP increase. The rectangular sensor was placed on the outside of the skull, and sensor readings were collected at each volume level. The VNA was calibrated to collect 5001 data points from 100 MHz to 1 GHz with a sweep duration of approximately 497 ms. Correlation between changes in volume and resonance frequency shifts were evaluated using the squared Pearson correlation coefficient.

To address limitations in the dry human skull model, a skin phantom was created and placed between the sensor and the skull (*ε_s_* ≈ 45). The phantom used in this study was a modified version of a white matter tissue phantom [32] altered to match the permittivity of skin and verified using a dielectric probe (DAK-12). The addition of this phantom adds a layer of complexity to the effective permittivity of the model and aimed to establish the ability of the sensor to function in a multi-layered, complex environment similar to that seen in the human body. Protocol similar to that used for the dry human skull model was used, with the amount of fluid injected into the system being increased from 150 mL to 200 mL and the increments being decreased from 10 mL to 5 mL.

### 2.4. Preliminary Proof-of-Concept Human Tests

#### 2.4.1. Human Subjects

Detecting shifts in intracranial pressure in the human body using the developed sensor requires the ability to detect fluid volume changes in a complex environment (human cranial cavity). To assess the sensor’s ability to detect fluid volume changes in the human cranial cavity, a preliminary head down tilt test was conducted with the rectangular sensor using a mannequin (control) and two healthy volunteers, one female (Participant 1; age: 20 years; height: 172 cm; weight: 79 kg) and one male (Participant 2; age: 19 years; height: 183 cm; weight: 64 kg). Additional testing was conducted to preliminarily assess the sensor’s ability to detect intracranial fluid volume changes induced by postural changes from upright to supine to head-down tilt. This second section of preliminary human tests was conducted with the square sensor using a mannequin (control) and four healthy male volunteers, (Participant 3; age: 19 years; height: 183 cm; weight: 64 kg, Participant 4; age: 23 years; height: 178 cm; weight: 68 kg, Participant 5; age: 21 years; height: 185 cm; weight: 99 kg, Participant 6; age: 21 years; height: 183 cm; weight: 84 kg). All participants were informed of the purpose and risks of the procedure and signed an informed consent form. The study protocols were approved by the Institutional Review Board (IRB) of Wichita State University. None of the participants had been diagnosed with a cardiovascular disease or other pathology affecting cerebral autoregulation, had undergone a revascularization procedure, or had implantable medical devices in their bodies.

#### 2.4.2. Validation of Bio-Fluid Shift

Simultaneous monitoring of the total intracranial fluid volume using MRI or intraventricular catheters would be optimal for validation, however, these methods require specific surgical expertise and equipment unavailable for this preliminary study. Therefore, the cross-sectional area of the internal jugular vein was used as validation of the cephalad bio-fluid shift in this proof-of-concept human study (without considering the correlation between sensor signal response and exact intracranial fluid volume). Axial views of the jugular vein were obtained using B-mode ultrasonography (Mindray M7, National Ultrasound, Duluth, GA, USA) and the DICOM file was used for analysis. Increases in intracranial fluid volume, such as those induced during postural changes (upright to supine or upright to head down tilt), have been associated with an increase in the cross-sectional area of the jugular vein [33]. As venous drainage increases in an attempt to return cerebral blood volume to the baseline level, the total blood volume in the jugular vein rises, driving an increase in cross-sectional area. Cross-sectional areas of the jugular vein were obtained using measurement tools onboard the ultrasound.

For further validation of the cephalad bio-fluid shifts, optic nerve sheath diameters were obtained and used to calculate estimated changes in ICP. Axial views of the optic nerve sheath were obtained using B-mode ultrasonography (Mindray M7, National Ultrasound, Duluth, GA, USA) and the DICOM file was used for analysis. As the pressure and volume in the cranial cavity increase, the increased CSF pressure in the subarachnoid space around the brain causes a swelling of the subarachnoid space around the optic nerve leading to an increase in the ONSD. Therefore, there is a direct relationship between ONSD and ICP. The ONSD measurements were used to quantify the induced change in ICP, using Equation (4) from literature [34].

(4) ICP=−111.92+(77.36×ONSD)

#### 2.4.3. Data Collection for Human Testing—Head Down Tilt Study

**Figure 3:**
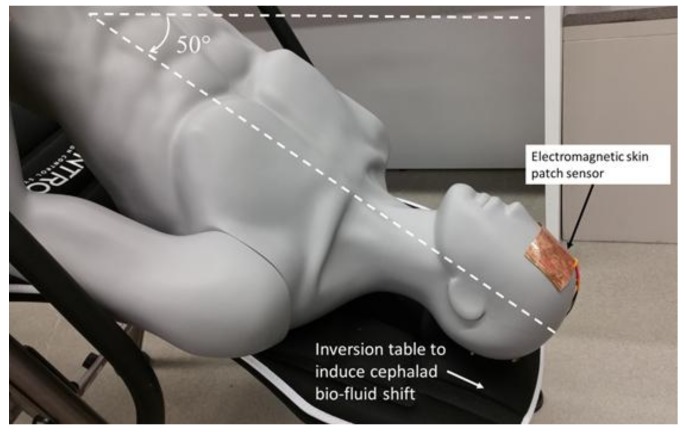
(Live human subjects were used—the mannequin provides visual representation only). The electromagnetic skin patch sensor to detect bio-fluid shift in 50° head down tilt. The mannequin was used to represent system set-up and sensor placement; however, data was collected from human participants.

An inversion table (Ironman Fitness iControl 600 Weight Extended Disk Brake System Inversion Table with Air Tech Backrest) was used to induce cephalad bio-fluid shifts (Figure 3). In the first stage of human testing, Participant 1 and Participant 2 were placed in two different angles (supine and 50° below supine) to induce the shifts in ICP due to changes in posture. The rectangular, electromagnetic resonant sensor was placed and secured on the participant’s forehead, and an RF sweep of 10 MHz to 3 GHz was used to find an optimal frequency for obtaining usable sensor signal response. The VNA was used to interrogate the sensor with an RF sweep from 1.75 GHz to 2 GHz and collect the S_11_ reflection coefficient of the first resonant frequency using 501 data points per sweep for approximately 70 s (as the fluid shift occurred). The sweeps began immediately after the participant was moved from supine to 50° below supine. Sweep data was extracted using Matlab 2016a and was smoothed using a 20-point moving-average filter. In each position, jugular vein cross-sectional area, systolic blood pressure, and diastolic blood pressure were measured.

In the second stage of human testing, the mannequin (control) and Participants 3–6 were positioned on the inversion table and moved between upright, supine, and 15° head down tilt to induce bio-fluid changes. This stage of preliminary testing was aimed at determining the ability of a smaller form factor sensor to detect induced intracranial bio-fluid shifts with the upright position as the baseline. To address the ability of a smaller sensor to detect intracranial bio-fluid shifts, the square sensor was adhered to the participant’s forehead. The patch was tested in several locations on the head besides the forehead in a small, pilot study. These locations include the parietal, occipital, and temporal regions of the head. With the experimental design utilizing an inversion table, the placement on the occipital region was not used due to the potential for varying pressure of the back of the head (with sensor attached) on the table which would result in increased variability during testing. The preliminary results of the pilot study indicated the greatest potential existing in sensor placement on the forehead. An RF sweep of 10 MHz to 3 GHz was used to find an optimal frequency for obtaining usable sensor signal response. The VNA was used to interrogate the square sensor with an RF sweep from 700 MHz to 1.1 GHz and collect the S_11_ reflection coefficient of the first principal resonant frequency using 5001 data points per sweep. Sensor readings were taken for each participant in the upright position, supine position, and every 90 s during a 30-min head down tilt at 15° below supine. Resonant frequency data was extracted using Matlab 2016a and smoothed using a 20-point moving-average filter. In each position, jugular vein cross-sectional area, systolic blood pressure, and diastolic blood pressure were measured.

#### 2.4.4. Repeatability of Measurements

Obtaining usable information regarding fluid volume detection requires the ability to obtain repeatable measurements, and during the human testing performed in this study, an important aspect to consider is the variance in sensor readings induced by changes in the participant’s posture. To evaluate the variation in the sensor’s resonant frequency, a preliminary repeatability test was conducted using a mannequin. The rationale for utilizing a mannequin for this repeatability study is to avoid the influence of day-to-day variations in human physiology on the assessment of the sensor’s variability. The impact of postural changes on shifts in the sensor’s resonant frequency was assessed by placing the square sensor on the forehead of a mannequin, and sensor readings were taken from the mannequin in the upright, supine, and 15° below supine positions. These readings were repeated every day for a total of three days with 10 samples in each reading. An analysis of variance (ANOVA) followed by a Bonferroni adjusted multiple comparison test was conducted to determine if postural changes had any significant differences in the resonant frequency shifts. Furthermore, the effect of temperature changes on shifts in resonant frequency was investigated. Sensor readings were obtained on a beaker of water at 35, 36, 37, 38, and 39 °C.

## 3. Results

### 3.1. Electromagnetic Resonant Sensor

**Figure 4:**
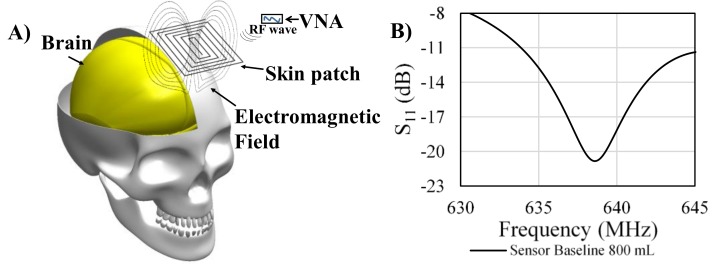
(**A**) The electromagnetic resonant sensor develops an electromagnetic field that surrounds the sensor; (**B**) The sensor resonates at different resonant frequencies depending on the substrate.

The passive spiral patch sensor was activated by gathering energy from the impinging RF wave. When the incident RF wave impinged upon the sensor, a current was induced in the spiral trace and produced electric and magnetic fields around the sensor which penetrated into the substrate, namely the skull (Figure 4A). At specific frequencies, the sensor resonated producing principal resonant frequencies and subsequent harmonic resonant frequencies (Figure 4B). The specific absorption rate for the system was calculated as approximately 0.114 W/kg.

### 3.2. Sensor Signal Response Due to Volumetric Changes in Skull Model

**Figure 5:**
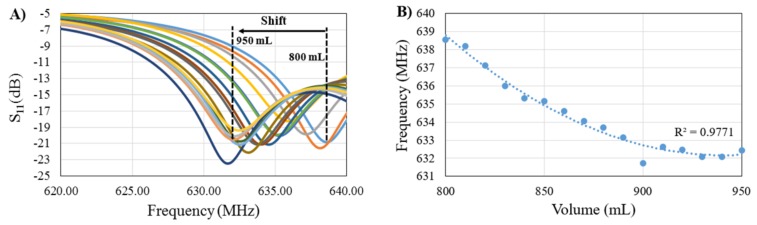
(**A**) Sensor signal response due to volume change. Each line color represents the sensor signal response at a different volume level; (**B**) Correlation between principal resonance frequency shift and volume change.

**Figure 6:**
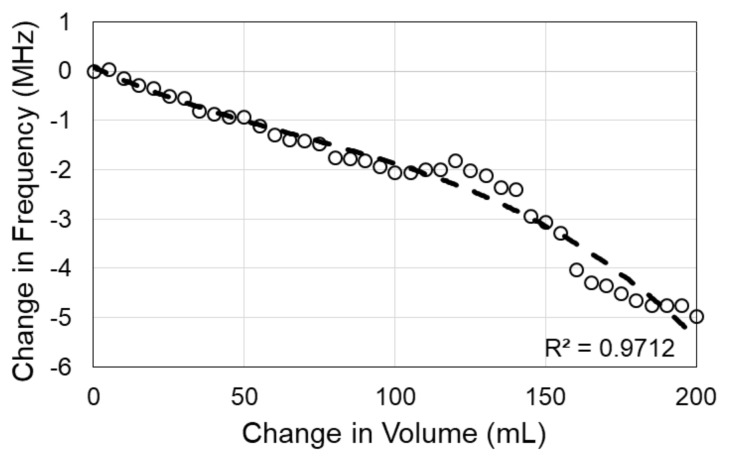
Correlation between principal resonance frequency shift and volume change in the model consisting of the dry human skull and head phantom.

Development of the physical model to mimic intracranial volume changes yielded useful data regarding the sensor’s ability to detect changes in fluid volume. Volume changes in the physical model triggered shifts in sensor signal response with 10 trials being conducted (Figure 5A). As the electromagnetic field propagated through the skull and fluid volume increased the fields were altered. These alterations prompted shifts in sensor signal response including a shift of the resonant frequency. Shifts in sensor signal response were primarily the result of changes in the effective electric permittivity of the layered system. Overall, fluid volume increases from 800 mL to 950 mL resulted in a net leftward shift in resonant frequency of 8.64 MHz. The standard error of the mean of the resonant frequency readings between the 10 trials at each volume was approximately 5.9 kHz. The shift in resonant frequency arising from the 10 mL increases in fluid volume had statistically significant differences (*p* < 0.01) for the first 13 volume levels, when using the Bonferroni adjusted multiple comparison test. The last three shifts in fluid volume (10 mL increments), from 920 mL to 950 mL, were not detected by the sensor. However, the 40 mL shift from 910 mL to 950 mL was detected. The correlation between the changes in principal resonance frequency of the sensor and the volume changes had an *R*^2^ = 0.97 (Figure 5B) and was classified as a second order polynomial. Furthermore, in the physical model consisting of the dry human skull and skin phantom, an overall fluid volume increase of 200 mL corresponded with a net leftward shift in resonant frequency of 4.97 MHz. The correlation between the changes in principal resonance frequency of the sensor and the volume changes in this more complex model had an *R*^2^ = 0.97 (Figure 6) and was classified as a third order polynomial.

### 3.3. Preliminary Human Tests

**Figure 7:**
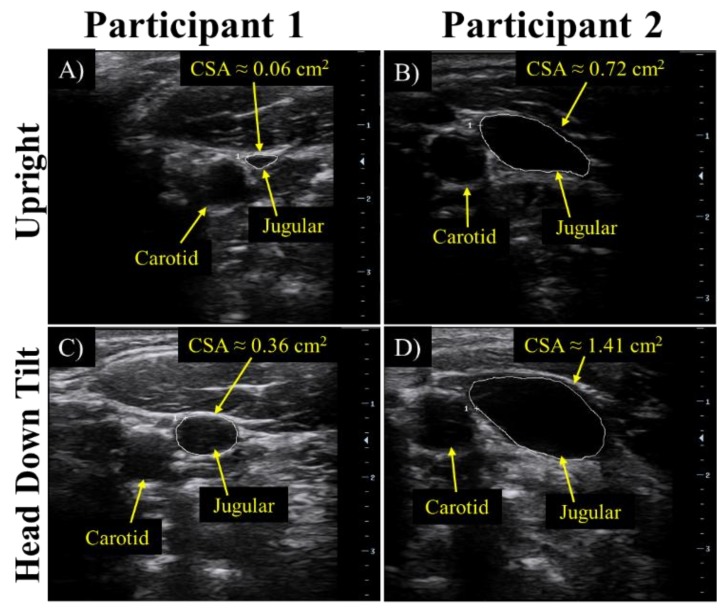
For the 70 s head down tilt at 50°, validation of the cephalad bio-fluid shift was determined by the cross-sectional area data obtained from ultrasound of the jugular vein as seen in upright position for (**A**) participant 1 and (**B**) participant 2, and for head down tilt for (**C**) participant 1 and (**D**) participant 2.

During a head down tilt test, the shift in bio-fluids from the lower body to the head and torso is accompanied by dilation of the internal jugular vein. Axial view ultrasound images of the internal jugular vein were used to validate the successful induction of an increase in intracranial fluid volume. During the first stage of human testing, in the upright position before the bio-fluid shift was induced, the cross-sectional area of the jugular vein, as calculated using the measurement tool onboard the ultrasound machine, was approximately 0.06 cm^2^ and 0.72 cm^2^ for participants 1 and 2, respectively (Figure 7A,B). During head down tilt, the cross-sectional area increased to approximately 0.36 cm^2^ and 1.41 cm^2^ for participant 1 and 2, respectively (Figure 7C,D).

**Figure 8:**
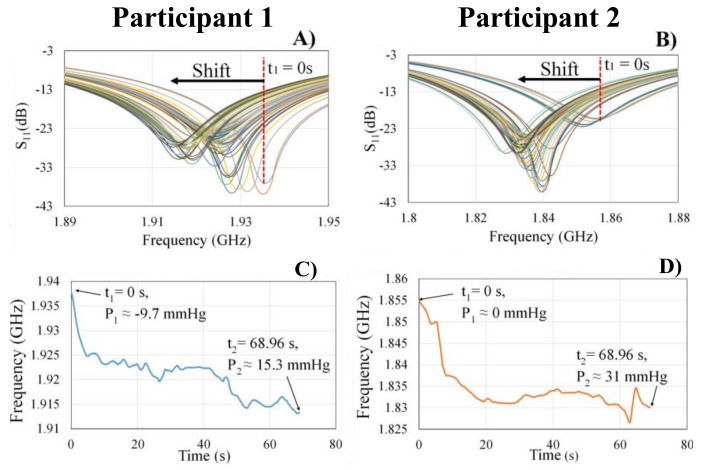
The sensor’s signal response corresponding with a cephalad bio-fluid shift was seen as shifts in the sensor’s principal resonant frequency in (**A**) participant 1 and in (**B**) participant 2. Each line color represents the sensor signal response at different times during the bio-fluid shift. The resonant frequency shift during an induced bio-fluid shift over time for (**C**) participant 1 and (**D**) participant 2.

Shifts in sensor signal response corresponding with the induced bio-fluid shift during the 70 s head down tilt were observed as seen in Figure 8A,B for participants 1 and 2, respectively. Over time, the fluid volume also corresponded with a negative shift of the resonant frequency (Figure 8C,D). The shift in the resonant frequency from time = *t*_1_ to time = *t*_2_ is approximately 24.4 MHz for participant 1 which corresponds to a 0.3 cm^2^ increase in jugular vein cross-sectional area. Whereas, a 24.825 MHz shift was observed in participant 2 with a corresponding cross-sectional area increase of approximately 0.69 cm^2^.

**Figure 9:**
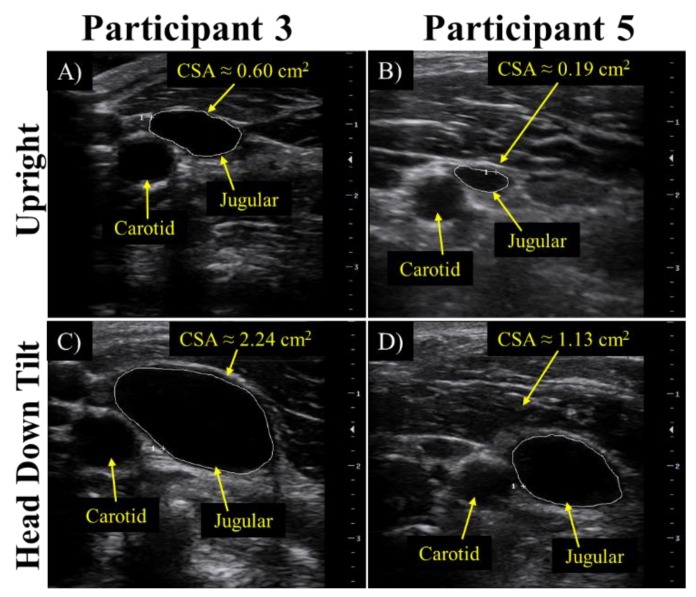
For the 30 min head down tilt at 15°, cross-sectional area data was obtained from ultrasound of the jugular vein as seen in upright position for (**A**) participant 3 and (**B**) participant 5, and for head down tilt for (**C**) participant 3 and (**D**) participant 5.

**Figure 10:**
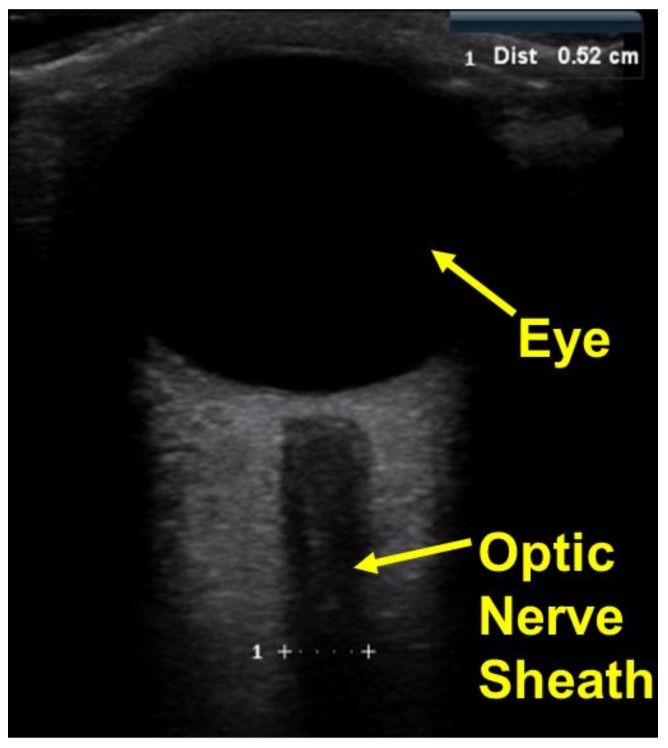
Optic nerve sheath diameter data was obtained from ultrasound imaging of the eye and used to calculate intracranial pressure (ICP).

**Figure 11:**
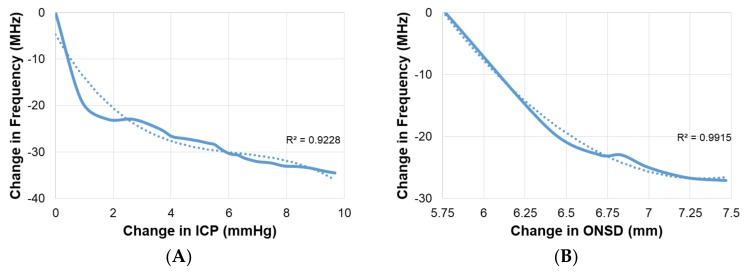
(**A**) Correlation between intracranial pressure (calculated using optic nerve sheath diameter) and principal resonant frequency shift; (**B**) Correlation between optic nerve sheath diameter and principal resonant frequency shift. The dotted lines are trend lines.

During the second stage of human testing, the increase in intracranial fluid volume was validated by observing the increase in venous outflow, indicated by an increase in the cross-sectional area of the jugular vein for the all participants, and the increase in optic nerve sheath diameter. The average dilation of the jugular vein from the upright position to the end of the 30-min 15° head down tilt was approximately 1.18 cm^2^. Representative figures of the dilation of the jugular vein can be observed in Figure 9. Whereas the average diameter increase of the optic nerve was approximately 1.7 mm. Using the diameter of the optic nerve, ICP estimates were obtained with an average change in ICP of approximately 9.67 mmHg. Representative figures of the optic nerve sheath can be observed in Figure 10. Correlation of the change in ICP vs. change in frequency and change in ONSD vs. change in frequency can be observed in Figure 11.

**Figure 12:**
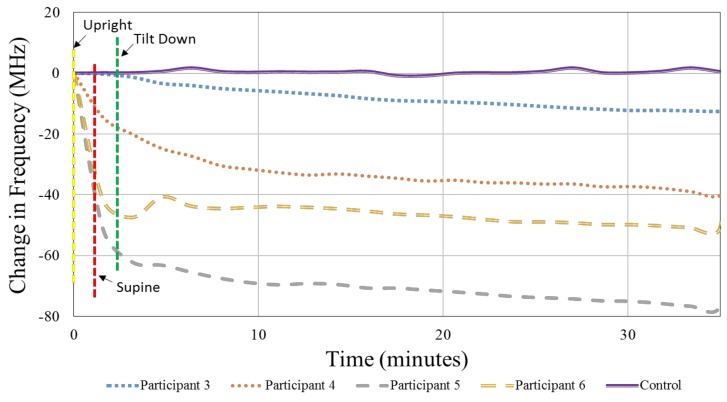
The sensor’s signal response corresponding with a cephalad bio-fluid shift was seen as shifts in the sensor’s principal resonant frequency for the 4 participants and the control.

Additionally, over the course of the 30-min head down tilt, the increased bio-fluid volume in the cranial cavity corresponded with a negative shift of the resonant frequency for all four human participants (Figure 12), whereas the control remained close to 0 (within ±1.2 MHz). Figure 12 represents the shifts in resonant frequency over time after a 20-point moving-average filter to smooth the data. The average shift in the resonant frequency from the upright position (time = 0 min) to the end of the 30 min head down tilt (time = 35 min) was approximately 45.07 MHz.

**Figure 13:**
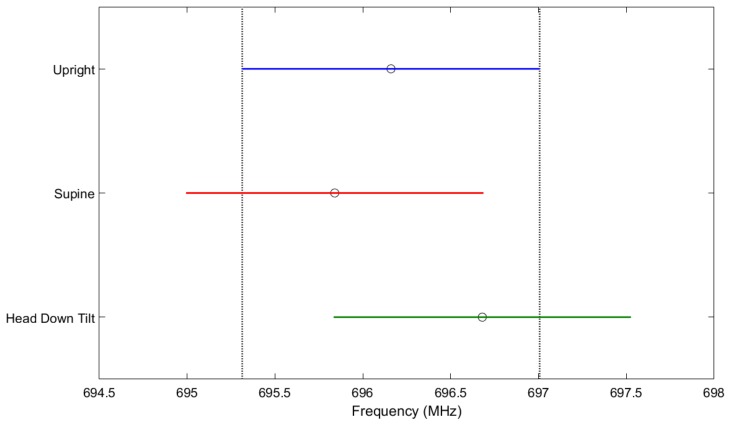
Repeatability test due to postural changes on a mannequin control. The sensor readings for the various positions had approximately a 2.5 MHz shift due to postural changes. When compared to the 45 MHz shift seen on a human due to bio-fluid shift, the shift due to postural changes were considered negligible. The blue line represents the variation of resonant frequency for the upright position, the red line represents the variation in supine, and the green line represents the variation during head down tilt.

Assessment of the repeatability of the sensor readings from the mannequin in multiple postures was obtained and statistically analyzed. For the three days, the average and standard deviations of the resonant frequency over ten sweeps in the upright, supine, and head down tilt positions were 696.16 ± 1.03, 695.84 ± 0.73, and 696.68 ± 1.08 (MHz), respectively. An ANOVA was conducted, followed by a Bonferroni adjusted multiple comparison test which indicated that there were not significant differences (*p* > 0.05) in resonant frequency resulting from postural changes, shown in Figure 13. Additionally, the change in temperature from 35 °C to 39 °C resulted in a total shift of less than 100 kHz. Significantly less than the magnitude of shift in resonant frequency due to increase in fluid volume.

## 4. Discussion

In this study, we have demonstrated the ability of an electromagnetic skin patch sensor to detect shifts in fluid volume of 10 mL through cranial bone. The ability of the sensor to detect volume changes of 10 mL is a robust step towards quantifying non-invasive intracranial fluid volume measurements. Although other non-invasive methods such as ultrasound possess the capability to detect volume changes, penetration depth through cranial bone has been a limiting factor. This sensor addresses the concern of penetration depth through the utilization of electromagnetic waves instead of the mechanical sound waves used in ultrasound. Electromagnetic waves permeate through structures, such as bone, more effectively than their mechanical counterparts, providing a larger range of clinical applications. Additionally, the SAR values obtained in this study (0.114 W/kg) is well within the accepted range resulting in minimal risk to the participants. Subsequently, this method to measure changes in fluid volume addresses three major limitations regarding intracranial pressure/volume measurement methods: invasiveness, penetration depth, and the need for specialized medical imaging equipment (MRI or CT).

The results of this study indicate that shifts in resonant frequency correspond with shifts in the effective permittivity of the system associated with increased bio-fluid volume in the cranial cavity. This volume detection capability may be utilized to indicate the presence of altered cerebrovascular function associated with volumetric increases in CSF and cerebral blood volume. 

### Potential Clinical Applications

This sensor technology may be harnessed as a point-of-care biomedical diagnostic to track the progression of diseases related to bio-fluid volume irregularities in the cranial cavity. A long term goal of this research is to utilize the effective electric permittivity of the blood, CSF, and brain tissue in the cranial cavity as a screening and monitoring tool for a variety of conditions including: hydrocephalus, hemorrhage, stroke, aneurysm, and edema. The optimization of a technology to be used to screen and monitor these conditions without the need for specialized equipment or training is particularly advantageous in low-resource settings such as the microgravity environment of space, rural communities, and for use by emergency medical services. Through the simplicity of the application and operation of our resonant sensor, several risks and limitations associated with current methods for measuring intracranial volume/pressure could be significantly reduced while opening up new possibilities.

Further optimization of the electromagnetic skin patch sensor may not only provide a point-of-care alternative to quickly monitor the risk of cerebral edema due to intracranial bleeding caused by brain trauma, but could also allow for assessment of severity of the injury. Previous studies have explored links between intracranial pressure increases and mechanisms affecting normal intracranial fluid dynamics such as traumatic brain injury [35,36]. Currently, standard imaging modalities such as CT and MRI lack the ability to be used for a confirmed concussion diagnosis, but are often used to monitor complications of the trauma including bone fracture and intracranial bleeding [7]. This technology is not widely available in limited resource settings and limits the ability for individuals in need to seek diagnostics and treatment [11,19]. The capacity to diagnose and monitor concussions and traumatic brain injuries is an area of interest in both the clinical setting and the realm of competitive sports. In many areas of athletics (particularly contact sports), pre-participation exams are already in place in many settings to establish an individual’s baseline as a safeguard to determine when they can return to normal activity following a concussion. Current practices help minimize complications such as second impact syndrome [7]. Although highly debated, this disorder is believed to occur when an individual receives a second injury before the initial injury heals. Due to autoregulation already being disrupted, this can lead to more serious damage including cerebral edema and herniation [37,38]. Alternative screening practices could be developed using this sensor technology to assist in the diagnosis of concussions along with the potential monitoring of the brain’s healing process, further reducing the risk of secondary complications.

Despite strong correlations between fluid volume changes and sensor signal response, the portion of the study using a simple model does possess several limitations. These include the simplification of the model, the use of only one human skull, and the absence of other biological tissue present in the layers of the body (skin, adipose, muscle, and brain tissue). The contents of the cranial cavity were simplified to be represented by water in a bladder. This simplification also implies a static fluid scenario which ignores the movement of blood and cerebrospinal fluid throughout the cranial cavity. However, these limitations were addressed through the incorporation of preliminary human tests. Implementation of human testing in this study assessed the sensor’s ability to detect fluid volume shifts, measured by shifts in resonant frequency, in a complex environment.

The primary limitation of human tests in this study is the number of participants in both the first stage and the second stage. Due to the small sample size, the analysis of the sensor’s ability to detect changes in bio-fluid volume is still preliminary. Despite the preliminary nature of this work, data collected throughout this study has indicated that the proposed sensor technology is able to detect induced volumetric increases in intracranial fluid volume. This detection capability is supported by the shift in the sensor’s resonant frequency corresponding to increases in intracranial fluid volume. An additional limitation is the inability to quantify the exact volume increase in the cranial cavity—which requires highly specialized medical imaging systems such as MRI. However, the use of ultrasound to track the cross-sectional area of the jugular vein provides evidence supporting the assumed increase in intracranial fluid volume. Future studies will look to (1) improve validation of the sensor’s performance through the use of phase contrast magnetic resonance imaging to quantify changes in CSF and cerebral blood volume and (2) increase sample size to provide for increased statistical analysis and evaluation of the sensor’s capabilities.

## 5. Conclusions

The electromagnetic resonant skin patch sensor developed in this study was able to detect fluid volume changes in not only a benchtop model, but also in preliminary human tests. This detection capability relies upon the sensor’s ability to detect alterations in the effective electric permittivity of a layered system. The results demonstrate an ability for the sensor to detect an increase in intracranial fluid, which is an integral step in creating an approach to non-invasively monitor shifts in intracranial pressure. This work combined with the optimization of the wearable form factor may also help identify novel approaches for the detection and monitoring of a variety of conditions including hydrocephalus, cerebral edema, concussions, and stroke.
